# AutoPET Challenge on Fully Automated Lesion Segmentation in Oncologic PET/CT Imaging, Part 2: Domain Generalization

**DOI:** 10.2967/jnumed.125.270260

**Published:** 2026-03

**Authors:** Jakob Dexl, Sergios Gatidis, Marcel Früh, Katharina Jeblick, Andreas Mittermeier, Anna Theresa Stüber, Balthasar Schachtner, Johanna Topalis, Matthias P. Fabritius, Sijing Gu, Gowtham Krishnan Murugesan, Jeff VanOss, Jin Ye, Junjun He, Anissa Alloula, Bartłomiej W. Papież, Zacharia Mesbah, Romain Modzelewski, Matthias Hadlich, Zdravko Marinov, Rainer Stiefelhagen, Fabian Isensee, Klaus H. Maier-Hein, Adrian Galdran, Konstantin Nikolaou, Christian la Fougère, Moon Kim, Nico Kallenberg, Jens Kleesiek, Ken Herrmann, Rudolf Werner, Michael Ingrisch, Clemens C. Cyran, Thomas Küstner

**Affiliations:** 1 Department of Radiology, LMU University Hospital, LMU Munich, Munich, Germany;; 2 Munich Center for Machine Learning, Munich, Germany;; 3 Department of Radiology, University Hospital Tübingen, Tübingen, Germany;; 4 Department of Radiology, Stanford University, Stanford, California;; 5 Comprehensive Pneumology Center, Member of the German Center for Lung Research, Munich, Germany;; 6 Konrad Zuse School of Excellence in Reliable AI, Garching, Germany;; 7 BAMF Health, Grand Rapids, Michigan;; 8 Shanghai AI Lab, Shanghai, China;; 9 Big Data Institute, University of Oxford, Oxford, United Kingdom;; 10 Université Rouen Normandie, LITIS UR 4108, Rouen, France;; 11 Nuclear Medicine Department, Henri Becquerel Cancer Center, Rouen, France;; 12 Siemens Healthcare SAS, Courbevoie, France;; 13 Karlsruhe Institute of Technology, Karlsruhe, Germany;; 14 HIDSS4Health, Karlsruhe and Heidelberg, Germany;; 15 Division of Medical Image Computing, German Cancer Research Center, Heidelberg, Germany;; 16 Helmholtz Imaging, German Cancer Research Center, Heidelberg, Germany;; 17 Pattern Analysis and Learning Group, Department of Radiation Oncology, Heidelberg University Hospital, Heidelberg, Germany;; 18 Universitat Pompeu Fabra, Barcelona, Spain;; 19 AIML, University of Adelaide, Adelaide, South Australia, Australia;; 20 Cluster of Excellence 2180, Image-Guided and Functionally Instructed Tumor Therapies, Tübingen, Germany;; 21 Department of Nuclear Medicine and Clinical Molecular Imaging, University Hospital Tübingen, Tübingen, Germany;; 22 German Cancer Consortium, Partner Site Tübingen, Tübingen, Germany;; 23 Institute for Artificial Intelligence in Medicine, University Hospital Essen, Essen, Germany;; 24 University Duisburg-Essen, Essen, Germany;; 25 Cancer Research Center Cologne Essen, University Medicine Essen, Essen, Germany;; 26 German Cancer Consortium, Partner Site Essen, Essen, Germany;; 27 Department of Nuclear Medicine, University Medicine Essen, Essen, Germany; and; 28 Department of Nuclear Medicine, LMU University Hospital, LMU Munich, Munich, Germany

**Keywords:** biomedical image analysis challenge, PET/CT, oncology, segmentation, deep learning, domain generalization

## Abstract

This article reports the results of the second iteration of the autoPET challenge on automated lesion segmentation in whole-body PET/CT, held in conjunction with the 26th International Conference on Medical Image Computing and Computer Assisted Intervention in 2023. In contrast to the first autoPET challenge, which served as a proof of concept, this study investigates whether machine learning–based segmentation models trained on data from a single source can maintain performance across clinically relevant variations in PET/CT data, reflecting the demands of real-world deployment. **Methods:** A comprehensive biomedical segmentation challenge on PET/CT domain generalization was designed and conducted. Participants were tasked to train machine learning models on annotated whole-body ^18^F-FDG data (*n* = 1,014). These models were then evaluated on a test set of 200 samples from 5 clinically relevant domains, including variations in institutions, pathologies, and populations and a different tracer. Performance was measured in terms of average dice similarity coefficient, average false-positive volume, and average false-negative volume. The best-performing teams were awarded in 3 categories. Furthermore, a detailed analysis was conducted after the challenge, examining results across domains and unique instances, along with a ranking analysis. **Results:** Generalization from a single-source domain remains a significant challenge. Seventeen international teams successfully participated in the challenge. The best-performing team reached an average dice similarity coefficient of 0.5038, a mean false-positive volume of 87.8388 mL, and a mean false-negative volume of 8.4154 mL on the test set. nnU-Net was the most commonly used framework, with most participants using a 3-dimensional U-Net. Despite competitive in-domain results, out-of-domain performance deteriorated substantially, particularly on pediatric and prostate-specific membrane antigen data. Detailed error analysis revealed frequent false-positives due to physiologic uptake and decreased sensitivity in detecting small or low-uptake lesions. A majority-vote ensemble offered minimal performance gains, whereas an oracle ensemble indicates hypothetical gains. Ranking analysis showed no single team consistently outperformed all others across ranking schemes. **Conclusion:** The second autoPET challenge provides a comprehensive evaluation of the current state of automated PET/CT tumor segmentation, highlighting both progress and persistent challenges of single-source domain generalization and the need for diverse public datasets to enhance algorithm robustness.

## Visual Abstract

**Figure:**
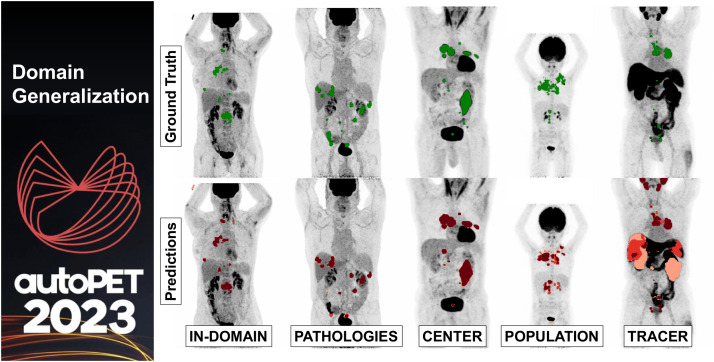

PET/CT plays a pivotal role in the diagnosis, staging, and treatment monitoring of cancer. Advances in machine learning (ML) have significantly improved the field of medical image analysis, enabling more precise segmentations across various imaging modalities. In oncologic imaging, the segmentation of active lesions offers the potential to improve downstream tasks such as lesion detection, characterization, and quantification, thereby directly supporting clinical decision-making (1).

Despite its importance, manual lesion segmentation is a time-consuming and subjective task (2–4). The urgent need for robust automated approaches was the motivating factor for the first autoPET challenge in 2022 (5). This first challenge demonstrated that ML models trained exclusively on ^18^F-FDG PET/CT data from a single institution performed well on in-domain evaluations. However, their performance declined when tested on an external dataset, underscoring the impact of domain shifts. Despite growing interest in ML-driven segmentation, comprehensive publicly available PET/CT datasets for deep learning remain limited, with few exceptions, such as autoPET (5,6) and HECKTOR (7). External validations of ML models are still rare and often fail to capture the sensitivity of these models to variations in scanner types, acquisition protocols, patient populations, and radiotracers. These domain dependencies demonstrate a major challenge for real-world deployment, particularly because most clinical centers lack the resources, expertise, or sufficient data to retrain or fine-tune models for their specific imaging protocols. This aligns with the technical task-specific evaluation in the Recommendations for Evaluation of Artificial Intelligence for Nuclear Medicine guidelines (8), which emphasize the importance of rigorous external validation for establishing the clinical utility of ML algorithms in nuclear medicine.

In response to these issues, we conducted the second edition of the autoPET challenge, which focused on single-source domain generalization (9)—a concept in which models are trained on a single-source domain and must perform well on previously unseen target domains without adaptation. Although the original training dataset of autoPET1 was used, this updated challenge features a more diverse test set that comprises 200 samples from 5 distinct domains, including different centers, pathologies, and patient populations and the use of a different radiotracer.

This paper presents the results of the autoPET2 domain generalization challenge hosted at the 26th International Conference on Medical Image Computing and Computer Assisted Intervention in 2023. We describe the challenge structure, test set composition, evaluation metrics, and especially methods used by participants, along with their performance. We analyze ranking stability, domain-specific performance, and how test data variability affects model performance. Overall, this study investigates the extent to which ML models can be developed to effectively segment lesions across multiple PET/CT domains without requiring domain-specific training data.

## MATERIALS AND METHODS

This retrospective study has been approved by the institutional review board (833/2020BO2, University Hospital Tübingen [UKT]). All subjects signed an informed-consent form.

### Whole-Body PET/CT Segmentation Challenge

A comprehensive biomedical segmentation challenge on PET/CT domain generalization was designed and presented at the 26th International Conference on Medical Image Computing and Computer Assisted Intervention in 2023, in collaboration with the European Society of Hybrid, Molecular, and Translational Imaging. The autoPET2 challenge was organized by a consortium of radiologists and data scientists from UKT, the Ludwig Maximilian University Hospital Munich (LMU), and University Hospital Essen (UKE). The challenge was conducted on the Grand Challenge platform (grand-challenge.org). Participants submitted containerized algorithms for standardized evaluation on a private multiinstitutional test set. Algorithms were made publicly available under a permissive open-source license. The challenge granted awards for best performance, domain robustness, and scientific and engineering contribution. This article adheres to the Biomedical Image Analysis Challenges guidelines for transparent reporting (10). Because of space constraints, we provide sections on the challenge mission, challenge organization and infrastructure, participation policies, and reference algorithms in the supplemental materials (supplemental materials are available at http://jnm.snmjournals.org).

### Datasets

An overview of the patient characteristics in the training and test sets can be found in Table 1. Distributions of SUV_mean_ and SUV_max_ per lesion can be found in Supplemental Figure 1. The training data include 1,014 anonymized whole-body ^18^F-FDG PET/CT scans from 900 patients diagnosed with cancer, each with manually annotated segmentation labels of metabolically active lesions. All scans were acquired at a single institution (UKT) using a Biograph 128 mCT PET/CT scanner (Siemens Healthineers). The training dataset was released as part of the autoPET1 challenge (7) and is publicly available via the Cancer Imaging Archive (11).

**TABLE 1.** Patient Characteristics of Training and Test Datasets

Characteristic	Training	UKT	UKT-Patho	LMU	UKE-Pediatric	LMU-PSMA
Dataset type	Training	Test	Test	Test	Test	Test
Institution location	Tübingen	Tübingen	Tübingen	Munich	Essen	Munich
Description	In-domain	In-domain	Different pathologies	Different institution	Different population	Different tracer
Patients	900	50	25	25	50	48
Studies	1,014	50	25	25	50	50
F/M sex	444/570	15/35	4/21	6/18	20/30	0/50
Age (y)	60 ± 16	55 ± 12	62 ± 17	56 ± 18	10 ± 6	71 ± 8
Weight (kg)	79 ± 19	92 ± 21	78 ± 20	72 ± 12	41 ± 29	82 ± 9
Scanners	1	1	1	4	1	4
Tracer	^18^F-FDG	^18^F-FDG	^18^F-FDG	^18^F-FDG	^18^F-FDG	^18^F-PSMA-1007 or ^68^Ga-PSMA-11
Studies with detectable lesions	513	31	25	25	37	25
Lesions	8,781	516	128	135	164	1,257
TMTV (mL)	110,189	7,127	1,206	3,859	5,189	7,332

TMTV = total metabolic tumor volume.

Data are number or mean ± SD.

The private test data consist of 200 PET/CT scans acquired at 3 institutions—UKT, LMU, and UKE—and describe 5 distinct imaging domains. The first subset, UKT (*n* = 50), consists of whole-body ^18^F-FDG PET/CT volumes drawn from the same institution and imaging distribution as the training dataset. The cohort includes patients (*n* = 50) diagnosed with lymphoma, non–small cell lung cancer, and melanoma, as well as negative cases.

The second subset, UKT-Patho (*n* = 25), was also acquired at UKT under similar imaging settings but with different primary tumors: esophageal carcinoma, colon cancer, anal cancer, pancreatic cancer, seminoma, breast cancer, cervical cancer, endometrial cancer, and rectal cancer.

The third subset (*n* = 25) was obtained at LMU. Scans were acquired on 3 Biograph scanners (128, 64, and 20 mCT; Siemens) and 1 Discovery 690 scanner (GE HealthCare). Primary diagnoses were lymphoma and non–small cell lung cancer.

The fourth subset, UKE (*n* = 50), consisted exclusively of pediatric patients, reflecting huge differences in age and weight distributions, and was acquired at UKE. Patients were diagnosed with lymphoma, lung cancer, sarcoma, hepatoblastoma, and melanoma; negative cases were also included.

The final subset, LMU prostate-specific membrane antigen (PSMA; *n* = 50), represents a substantial domain shift due to the use of a different tracer—either ^18^F-PSMA-1007 or ^68^Ga-PSMA-11. It contains whole-body PSMA PET/CT scans from LMU. The cohort consists of patients with suspected or diagnosed prostate carcinoma. Scans were performed on the same 4 scanners as the LMU subset.

The test set was labeled using the same procedure as for the curated training dataset (6).

### Challenge Participation and Submitted Algorithms

Challenge statistics were collected through the Grand Challenge platform: information about participation, team formation, submissions, and geographic distribution of participants. A retrospective analysis of algorithmic approaches submitted by participants to the challenge was conducted. Methodologic details were primarily extracted from associated preprints. In cases of ambiguity, such as submissions with multiple models or unclear configurations, additional information was inferred from submitted Docker container logs and available code repositories (https://www.docker.com/).

### Performance of Submitted Algorithms

Segmentation performance of the submitted algorithms was evaluated using the overall dice similarity coefficient (DSC), the false-positive volume (FPV), and the false-negative volume (FNV), which are defined in the supplemental materials. These metrics were chosen to maintain consistency with the previous challenge iteration (5), enabling direct comparison across studies. Participants were ranked based on the average performance across all 3 metrics, with the DSC weighted twice to reflect its importance in lesion segmentation accuracy. The best-performing teams were awarded in 3 categories: award category 1 for overall performance, award category 2 for robustness across domains, and award category 3 for outstanding scientific or engineering contributions (supplemental materials).

In addition, per-domain performance was evaluated using the DSC, FNV, and FPV. The distribution of the mean performance of all teams was analyzed. Out-of-the-box nnU-Net (Division of Medical Image Computing, German Cancer Research Center) (12) was trained and used as the baseline model (supplemental materials).

### Lesion Detection Sensitivity

Per-lesion sensitivity was analyzed in relation to lesion volume, as well as SUV_mean_ and SUV_max_. For that, lesions were grouped in interdecile ranges and then split by dataset domain to reveal systematic errors. Sensitivity is calculated based on the FNV criteria (i.e., the lesion was considered detected if at least 1 pixel was identified), which were evaluated across all teams.

### Ensembling

To explore the potential of combining top-performing algorithms, ensemble methods were applied. A majority-vote ensemble model was constructed from the algorithms ranked in award category 2, including the baseline. In addition, an oracle ensemble was simulated by selecting the highest-performing prediction for each case based on the DSC, representing the upper bound of the challenge performance. This does not reflect a true theoretic bound but instead provides an indication of the maximum performance achievable for each case given the available submissions, thereby offering insight into dataset noise and case difficulty.

### Qualitative Error Analysis of Algorithm Performance on Test Data

To further evaluate the impact of the test data on the algorithm performance, an error analysis was conducted in terms of the performance metrics. The distribution of the performance metrics over the individual case was assessed across all teams. Test case predictions were also manually reviewed on the basis of their maximum-intensity projection, and common segmentation errors were qualitatively analyzed.

### Statistical Analysis

Analyses were conducted after the challenge to assess the robustness and interpretability of the results. Ranking stability was evaluated using bootstrap resampling (*n* = 1,000) to assess the robustness of the leaderboard under sampling variability (13). In addition, alternative ranking approaches were explored to provide different perspectives on team performance. These included median rank, rank-then-aggregate, and subgroup-wise mean ranking (supplemental materials). We also compared the average results of the autoPET1 and autoPET2 participants on the identical sets of test cases from the UKE and LMU data.

## RESULTS

### Challenge Participation and Submitted Algorithms

As of November 2024, 344 individuals had registered for the autoPET2 challenge, resulting in the formation of 26 teams. The participants were predominantly concentrated in Asia (59%, with 39% from China), followed by Europe (26%, primarily Germany at 6%) and North America (12%, mainly the United States at 9%).

During the preliminary phase, 37 teams participated, submitting 218 algorithms. In the final phase, 17 teams submitted 27 successful algorithms, and the highest-performing submission from each team was used to determine the final leaderboard. One team (Blackbean) submitted 4 algorithms, 7 teams (zstih, agaldran, cv:hci, anissa218, Y. Chen-PET-MiLab, IMIT-RJH, and ahnssu) submitted 2 algorithms, and the remaining 9 teams submitted just 1 algorithm. The ranking is based on all submissions.

Most teams used 3-dimensional U-Net architecture (13/17), with alternatives including SegResNet, 2-dimensional U-Nets, hybrid 2-dimensional and 3-dimensional models, and transformer-based architecture. Automated frameworks such as nnU-Net (8/17) (12) and MONAI’s Autoseg3D (1/17) (14) were frequently used. Nearly all participants incorporated both CT and PET volumes, primarily handling multimodality through concatenation. Two teams used multibranch networks. A multistage design in which samples are staged for segmentation by a discriminator network was used by 3 teams. Organ segmentation labels were incorporated by 3 teams, either end to end or for pretraining. Labels were generated using TotalSegmentator (Department of Research and Analysis, University Hospital Basel) (15) and MOOSE (ENHANCE-PET Research Group) (16). Many teams tweaked nnU-Net configurations, including different loss functions, backbones, training hyperparameters, and postprocessing strategies. Dice with cross-entropy was most common (11/17). Test-time augmentation, connected component removal, and ensembling (often 5-fold cross-validation) were widely used. Hardware setups varied substantially, ranging from consumer graphics processing units to multi-A100 systems (Nvidia). A detailed summary of the submitted algorithms can be found in the supplemental materials, accompanied by Supplemental Table 1.

### Performance of the Submitted Algorithms

**FIGURE 1.**
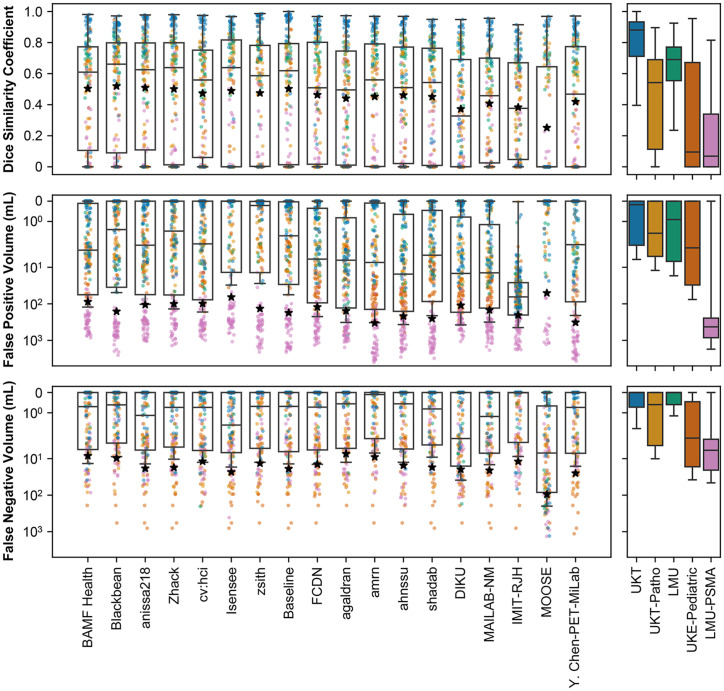
Distribution of results of all ranked algorithms on all 3 evaluation metrics. Order from left to right signifies final leaderboard of award category 1. Star symbolizes average performance, which was used for ranking. Right part of figure shows spread of performance over all samples, broken down by test subset.

Figure 1 depicts the performance of the 17 submitted algorithms ordered by leaderboard position. The presented results show only the highest-ranked algorithm per team. However, ranks are calculated with respect to all algorithms. The complete ranking and leaderboard can be found in Supplemental Table 2.

Team Blackbean achieved the highest DSC (0.5198) but ranked second overall because of higher FPV (161.3918 mL, rank 14). First-ranked team BAMF Health showed balanced performance, with a DSC of 0.5038 (rank 5), FNV of 8.4154 mL (rank 3), and FPV of 87.8388 mL (rank 3). The baseline algorithm achieved a competitive DSC (0.5024, rank 6) but was limited by higher FNV (18.9489 mL, rank 19) and FPV (176.43 mL, rank 18), with 7 teams outperforming it. The median of all average DSCs was 0.4622 (range, 0.2515–0.5297). Average FNV ranged from 7.3769 to 95.2065 mL (median, 13.8600 mL), and average FPV ranged from 50.6383 to 378.1262 mL (median, 165.2457 mL).

Table 2 presents the stability metrics of award category 2 for the teams achieving a higher average DSC than the median of all submissions (supplemental materials). Blackbean demonstrated the highest DSC stability (1.5032). BAMF Health showed exceptional FNV stability (182.5391 mL) and placed first overall (combined rank, 1.75). Team Isensee achieved the most stable FPV (9,848.7539 mL), whereas the reference model (baseline) placed eighth, with particular weakness in FPV stability (76,533.4919 mL, rank 8). The best engineering contribution was awarded to team Isensee, and the best scientific contribution was given to team agaldran (award category 3).

**TABLE 2.** Final Leaderboard of Award Category 2 for Most Stable Performance Metrics

Position	Team	Combined rank	DSC	FNV (mL)	FPV (mL)
1	BAMF Health	1.75	1.4854 (2)	182.5391 (1)	15,695.4972 (2)
2	Blackbean	3	1.5032 (1)	501.1262 (3)	60,298.2747 (7)
3	cv:hci	3.25	1.3809 (4)	371.3454 (2)	19,315.1542 (3)
4	anissa218	4.25	1.4364 (3)	1,555.6952 (6)	23,938.1009 (5)
5	Zhack	4.75	1.3744 (5)	1,482.2795 (5)	23,587.6349 (4)
6	Isensee	5.75	1.3285 (7)	2,123.5454 (8)	9,848.7539 (1)
7	zstih	6.5	1.2773 (8)	723.3943 (4)	46,443.9119 (6)
8	Baseline	6.75	1.3706 (6)	1,597.9765 (7)	76,533.4919 (8)

Data are mean divided by SD followed by rank in parentheses. Only teams that achieved higher average DSC than median of all submissions were eligible. Mean of performance metrics were normalized by their respective SD over test set to obtain stability metrics.

**FIGURE 2.**
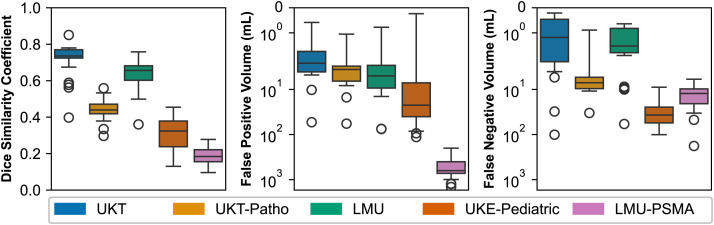
Distribution of mean performance of participating teams. Higher placement indicates better performance in respective metric (note inverted ordinate axis for FPV and FNV).

Figure 2 illustrates the distribution of mean performance per team across data domains, demonstrating the impact of domain variation on performance. The average DSC on the in-domain UKT dataset shows strong performance for most teams (median, 0.7355; maximum, 0.8509) and a narrow interquartile range of 0.0456. In contrast, the UKE-Pediatric dataset exhibits lower performance (median, 0.3239; maximum, 0.4547) and higher variance (interquartile range, 0.1410), suggesting considerable performance differences among teams in this domain. The LMU-PSMA dataset exhibited the lowest median DSC (0.1840), with a maximum of 0.2779 and a relatively small interquartile range of 0.0661, indicating consistently lower performance across teams for this subset.

The FPV for the LMU-PSMA dataset is orders of magnitudes larger (median, 630.1645; minimum, 200.0330). The UKE-Pediatric dataset has the next-largest average FPV (median, 22.3353; minimum, 0.2354; maximum, 113.7095).

With regard to FNV, most algorithms achieved relatively low averages across the datasets. However, the UKE-Pediatric dataset displayed the largest FNV (median, 36.8663) and a large interquartile range of 30.4265 mL. Detailed performance metrics for each team per domain can be found in Supplemental Tables 3–5.

### Lesion Detection Sensitivity

**FIGURE 3.**
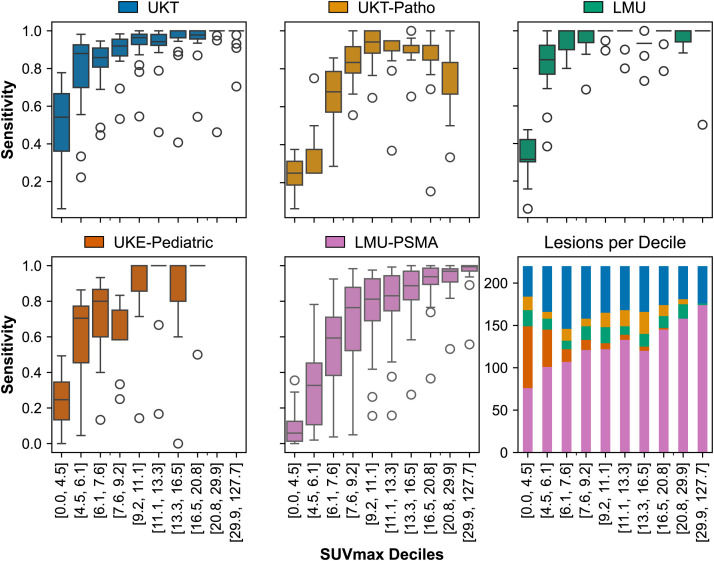
Distribution of lesion sensitivity and lesion count per test subset (domain) over all participating algorithms. Abscissa indicates SUV_max_ deciles, which were calculated over all lesions for comparability. Clearly visible is that lesions with lower SUV_max_ are detected less often across all domains.

Generally, smaller lesions are less frequently detected for all domains (Supplemental Fig. 2). Similarly, lesions with a lower SUV_mean_ (Supplemental Fig. 3) and SUV_max_ (Fig. 3) are detected less often. With consideration of the differing lesion distributions across datasets, it becomes apparent that, for example, the UKE-Pediatric dataset contains a higher proportion of such low-uptake lesions. A similar drop in detection is seen in the LMU-PSMA dataset, although this is less reflected in the average sensitivity metrics because of a greater number of high-uptake lesions compensating for it.

### Ensembling

A simple majority-vote ensemble was produced based on all algorithms, which are ranked in award category 2. The ensemble showed a DSC of 0.5170, FPV of 138.7576 mL, and FNV of 18.4111 mL. This ensemble does not outperform the single best-performing submissions. Through consideration of an oracle ensemble of all algorithms, that is, always choosing the best segmentation of all submissions with regard to the DSC, an average DSC of 0.6466 and an average FNV of 4.2586 mL were achieved. Furthermore, if this ensemble would segment only volumes with lesions, it would have an average FPV of 50.8864 mL. When we examine each test set domain individually, the performance gap between the best single algorithm and the oracle ensemble varies considerably. The in-domain (UKT) data have a relatively low potential average DSC improvement of 0.8509 (baseline) to 0.8770. LMU-PSMA from 0.2779 (Isensee) to 0.3243 and LMU 0.7584 (Isensee) to 0.8049 have mediocre potential. The highest improvements could be achieved for the UKE-Pediatric dataset, with 0.4465 (Blackbean) to 0.5466, and UKT-Patho, with 0.5329 (Blackbean) to 0.6731.

### Qualitative Error Analysis of Algorithm Performance on Test Data

**FIGURE 4.**
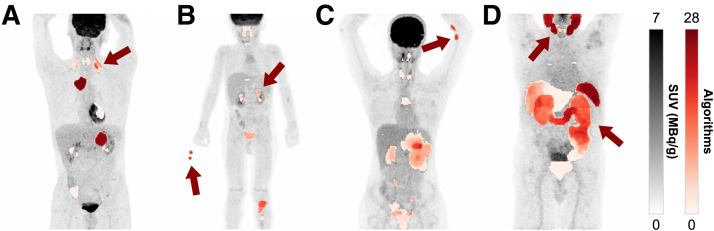
Common observed error patterns in algorithm predictions. (A–D) Segmentation errors due to muscle activity (A) with extracorporeal radiotracer contamination and in urinary tract of pediatric cases (B), paravenous extravasation (C), and physiologic uptake in salivary and lacrimal glands, bowel, bladder, liver, spleen, and gallbladder (D). Images show SUVs (center SUV, 3.5; window SUV, 7) alongside predictions of all submitted algorithms (darker colors indicate agreement among more algorithms, *n* = 28).

Visual inspection of maximum-intensity projections revealed several recurring error patterns of automated segmentations across submissions (selection in Fig. 4). Several test cases contained small, isolated lesions (1–10 voxels), challenging algorithm performance. Minor oversegmentation near PET/CT volume boundaries was sometimes observed, likely due to edge-related reconstruction artifacts. Nonpathologic uptake, such as physiologic muscle activity, was occasionally misclassified (Fig. 4A), whereas extracorporeal radiotracer contamination (Fig. 4B) and paravenous extravasation (Fig. 4C) were frequently misclassified.

In the LMU-PSMA dataset, most algorithms segmented physiologic uptake in the salivary and lacrimal glands, bowel, bladder, liver, spleen, and gallbladder (Fig. 4D). The top-performing model (BAMF Health), which incorporated organ priors, reduced abdominal false-positives but still mislabeled glandular tissue regularly. However, similar reductions were noted for the scaled nnU-Net approach (Isensee) with the lowest FPV.

In the UKE-Pediatric dataset, physiologic uptake in the urinary tract (Fig. 4B) was common, and uptake in the thymus, pharyngeal lymphoid tissue, vocal cords, and heart occurred regularly. Brown adipose tissue and bone marrow were sometimes segmented, likely reflecting immune activity after treatment.

We also looked at patient level and observed algorithm performance per patient (Supplemental Fig. 4). In the supplemental figure, a whisker for each patient in the test set is plotted with the prediction of every submitted algorithm. Clearly visible is a regime of patients (left side of the plot) for which no contestant achieved a reasonable performance. Of 200 patients, 20 have a maximum DSC smaller than 0.1000, reducing the average performance of participating algorithms. These samples featured small, singular lesions (Supplemental Fig. 5); lesions with low uptake; or rare tumor locations (i.e., in the brain or bladder).

### Statistical Analysis

**FIGURE 5.**
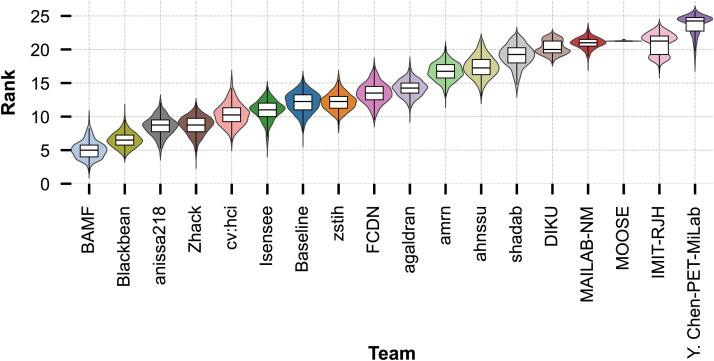
Ranking stability of participating teams based on 1,000 bootstrap iterations. Violin plots show distribution of ranks per team. Overlap of distributions of multiple teams highlights absence of single dominant model and emphasizes close competition among top-performing models.

To assess ranking stability, we performed a bootstrap analysis with 1,000 iterations, generating the ranking distributions for each team depicted in Figure 5. The results reveal a high degree of overlap across the teams, indicating that no single team consistently outperformed all others across the resampled datasets. For example, although BAMF Health achieved the best average performance (median rank, 4.96), its distribution overlaps with those of other top contenders, such as Blackbean, anissa218, and Zhack. Even midranked teams such as Isensee or our baseline show considerable overlap with teams ranked slightly above or below them.

To further explore how different ranking perspectives might influence the final leaderboard, we applied 3 alternative ranking strategies: median then rank, rank then aggregate, and average per subgroup then rank. As shown in Supplemental Figure 6, each method emphasizes slightly different performance aspects, leading to shifts in team rankings.

We also performed a direct comparison between the autoPET1 and the autoPET2 challenges by analyzing performance on the shared test subsets from the UKT and LMU datasets. As shown in Supplemental Figure 7, the performance distributions across both challenges are similar, suggesting consistency in model performance across iterations. AutoPET2 submissions exhibited slightly higher median and maximum DSC values on the LMU subset, pointing to a modest improvement in performance from the previous challenge.

## DISCUSSION

In this work, we present the results of the autoPET2 domain generalization challenge, which was held at the 26th International Conference on Medical Image Computing and Computer Assisted Intervention in 2023. The goal of autoPET2 was to explore the generalizability of deep learning models across clinically relevant, unseen domains and to further investigate the strengths and limitations of current segmentation algorithms for PET/CT imaging.

The challenge results highlight the difficulty of achieving robust domain generalization from a single training source. No single model or team consistently outperformed others across all datasets or evaluation metrics. Instead, performance was highly domain-dependent. The in-domain data (UKT) and LMU ^18^F-FDG data showed generally good segmentation quality, whereas performance declined for the UKT-Patho dataset and dropped substantially for the UKE-Pediatric and LMU-PSMA datasets. The best-performing method reached an average DSC of 0.5038, a FNV of 8.4154 mL, and a FPV of 87.8388 mL—indicating only moderate segmentation quality even among top submissions.

When we delve deeper into why performance varies so heavily across domains, systematic errors become evident. A dominant factor is the high FPV in the LMU-PSMA data, where values were up to 2 orders of magnitude larger than in other domains, which causes the FPV ranking to be dominated by the PSMA data. This is because PSMA samples have, as anticipated, a very different physiologic uptake pattern (17). Although ^68^Ga-PSMA-11 and ^18^F-PSMA-1007 exhibit distinct uptake patterns—diffuse bone versus bladder uptake—we did not observe significant visual differences in the test set that could affect the segmentation algorithms. Common segmentation errors include the abdomen, salivatory and lacrimal glands, bladder, liver, gallbladder, and spleen. Surprisingly, lesion sensitivity and the true positive metabolic tumor volume remained high in this dataset, indicating that most lesions are correctly detected.

The ^18^F-FDG UKE-Pediatric data seem to be more challenging. This dataset showed greater variance in team performance, suggesting higher inherent difficulty. It has substantial FPV in the urinary tract as well, sometimes in the thymus, lymphatic tissue of the pharyngeal area, vocal cords, heart, brown fat tissue, or bone marrow reactivation. In addition, the lesion detection sensitivity on this dataset is low, even lower than the LMU-PSMA data. This may be attributed to the different uptake pattern and lesion size distributions.

The lesion sensitivities of all algorithms are generally worse for very small lesions and for lesions with a low uptake (SUV_mean_ and SUV_max_). This trend is well documented in prior PET/CT segmentation studies, where smaller lesions were less reliably detected (5,18,19) and low-uptake lesions remained a persistent challenge for deep learning models (20). The UKE-Pediatric data comprise a larger proportion of such lesions, thus reducing the average performance.

To the question of whether any of the proposed methodologic advancements lead to better performance, we cannot give a definite answer. Despite the competitive nature of the challenge, no single method emerged as a clear winner. Bootstrap-based ranking analysis revealed significant overlap in team performance distributions, with even the top-ranked teams sharing broad rank intervals. In addition, alternative ranking strategies caused noticeable shifts in leaderboard positions, emphasizing the sensitivity of rankings to the evaluation procedure. The substantial technical challenge remains the reduction of false-positives while maintaining high lesion sensitivity. This was tackled in different ways by participating teams. One promising method is to use prior knowledge about physiologic uptake patterns via organ segmentations. The organ labels conditioned the model in training on regions with high physiologic uptake. In the results of the top-performing teams, we can also observe standard nnU-Net models, which achieve a smaller FPV (at the cost of a higher FNV). This also highlights that model calibration is difficult and might be worth investigating. Another interesting idea is to use a multistage approach, in which a discriminator network first decides whether an incoming volume contains pathologic uptake and, if it does, uses a second segmentation model. However, these methods did not translate well in terms of performance. For 1 team, this concept generally failed to make improvement against the sole segmentation network. For the other team, the discriminator failed to pass the correct samples to the second stage, dragging the performance. In a multidomain setting in particular, we think it is hard to ensure that the discriminator works as expected. Overall, many participants used the nnU-Net framework, sometimes with only minor adaptations such as a longer training time, and these participants still ranked among the top teams. This reinforces nnU-Net’s strength as a baseline method while illustrating the significant challenge of surpassing its performance in PET/CT segmentation tasks.

In a comparison with the same test data subsets from the autoPET1 challenge, no substantial improvement in performance for the UKT and LMU domains is observed, which may suggest a technical limit. However, we argue that this is not the case. An analysis of the submissions indicates that an optimal ensemble of the existing methods already increases the average DSC from the top-performing team’s score of 0.5297–0.6466, highlighting potential for further performance gains. Performance increases are also possible for the in-domain data, but to a lesser extent.

When we consider the clinical relevance of our results, cross-tracer generalization is exploratory, and in practice, a supervised, tracer-specific, or multitracer approach would generally be preferred. Nevertheless, the study of single-source domain generalization provides several clinically meaningful insights. First, it highlights how models behave under realistic distribution shifts, revealing robustness and potential failure modes that may be invisible in in-domain evaluations. Second, our experiments show that lesion sensitivity remains high even on PSMA cases, suggesting that models trained on a single source could assist lesion identification and accelerate the creation of annotated local datasets. Because experts are allowed to focus on reducing false-positives, this approach could be applied iteratively, saving substantial time and resources, as demonstrated by Wasserthal et al. (15).

A clear limitation of the challenge is the simple detection criteria of the FPV and FNV. Under the current scheme, even a single voxel overlap is sufficient to count a lesion as detected, effectively disregarding how well the predicted volume matches the annotated ground truth. This discrepancy is especially relevant when lesion volume is used as a clinical marker or when interpreting lesion detection sensitivity. Although the current criteria are coarse, they still offer a consistent and reproducible basis for benchmarking segmentation performance and provide complementary insights beyond the DSC (21). Compared with the nomenclature of Carass et al. (22), only detection failures are captured, without accounting for merge, split, and split–merge events. Aggregation by volume rather than by count weights each detection failure according to the size of the missed lesion, which represents a deliberate design choice. Expansion of the criteria in future versions could help capture more nuanced error types.

Another limitation arises from the variability of the reference annotations. We think that the test lesion segmentations are subject to substantial reader variability, as indicated in the autoPET1 challenge and others (3,5). This is especially true because the ground truths are produced by a morphologic reading from 3 different experts without a strict SUV threshold. This represents a potential source of annotation domain shift that may influence model generalization and is likely a substantial component of what is commonly called a center shift. A drawback of the current evaluation framework is that this variability has not been quantified for the new test sets. Finally, it would be valuable to disentangle the contribution of individual domain shift factors (scanner, patient population, tracer, and annotation). The current challenge design emphasizes single-source domain generalization and therefore captures the aggregate effect of these factors, because they naturally cooccur in real-world clinical settings. Thus, the results should be interpreted as reflecting the combined impact of multiple, interacting sources of variability, rather than isolated effects. Future studies could aim to quantify these contributions individually.

## CONCLUSION

The autoPET challenge series aims to facilitate progress in automated PET/CT analysis by motivating research, enabling algorithm benchmarking, documenting advancements, and providing large machine-ready datasets. AutoPET2 provides a comprehensive evaluation of the current state of automated PET/CT tumor segmentation, highlighting both progress and persistent challenges in the field. Single-source domain generalization remains a challenge for whole-body PET/CT segmentation, and ML algorithms are not yet ready for direct application in diverse clinical settings. However, most domain discrepancies often stem from simple systematic errors, and teams achieve strong in-domain performance. In addition, most algorithms are able to detect a large fraction of lesions, and the major obstacle lies in the reduction of false-positives. This underscores the pressing need for more diverse public PET/CT datasets to support robust ML development. Future challenges will address these limitations.

## DISCLOSURE

Financial support of the study was provided through the Adolf-Leuze foundation. The work was supported by the Deutsche Forschungsgemeinschaft under Germany’s Excellence Strategy EXC 2064/1 (project number 390727645) and EXC 2180 (project number 390900677). This paper is supported by the DAAD programme Konrad Zuse Schools of Excellence in Artificial Intelligence, sponsored by the Federal Ministry of Research, Technology and Space. The authors gratefully acknowledge LMU Klinikum for providing computing resources on their Clinical Open Research Engine (CORE). We disclose that some authors have industry affiliations. However, no industry sponsorship or industry support was obtained for this research. The corresponding academic authors had full control over the data and their analysis. No other potential conflict of interest relevant to this article was reported.

## Notes

KEY POINTS **QUESTION:** Can ML-based segmentation models, trained on data from a single source, maintain performance across clinically relevant variations in PET/CT data? **PERTINENT FINDINGS:** Performance was highly domain-dependent and challenging for all algorithms. **IMPLICATIONS FOR PATIENT CARE:** Generalization of segmentation algorithms will provide support for clinicians quantifying prognostic markers. If robustness is ensured, it will help to reduce fatal prediction errors.
